# Usability of therapy controllers in elderly patients with deep brain stimulation

**DOI:** 10.1186/1471-2377-12-4

**Published:** 2012-02-13

**Authors:** Iris Kaiser, Ilse Kryspin-Exner, Thomas Czech, Francois Alesch

**Affiliations:** 1 Department of Neurosurgery, Medical University of Vienna, Währinger Gürtel 18-20, 1090 Wien, Austria; 2 Institute of Clinical, Biological and Differential Psychology, Faculty of Psychology, University of Vienna, Liebiggasse 5, 1010 Wien, Austria

## Abstract

**Background:**

Technical devices are becoming more prevalent in society and also in medical care. Older adults need more support to learn new technologies than younger subjects. So far, no research has been done on the usability of patient controllers in deep brain stimulation in an elderly population. The aim of the study was to investigate the factors influencing the performance of elderly DBS patients with respect to usability aspects of Medtronic Access therapy controllers.

**Methods:**

Time, mistakes and frequency of use of the controller were compared in 41 elderly DBS patients who prior to the study had already owned a therapy controller for more than six years. One group (n = 20, mean age = 66.4 years) was watching an instructional video and then completed practical assignments on a model implantable pulse generator (IPG). The other group (n = 21, mean age = 65.9 years) completed the tasks without having seen the video before. Any errors that patients made were documented and also corrected so that all of them received hands-on training. After six months all patients were re-evaluated on the dummy IPG in order to compare the effects of hands-on alone vs. video-based training combined with hands-on.

**Results:**

The group that had seen the video before significantly outperformed the control group at both assessments with respect to number of errors. Both groups performed faster after six months compared to baseline and tend to use the controller more often than at baseline.

**Conclusion:**

Our results indicate that elderly DBS patients who have been using the controller for several years still have various difficulties in operating the device. However, we also showed that age-specific training may improve the performance in older adults. In general, the design of DBS patient controllers should focus on the specific needs of the end-users. But as changes to medical devices take a long time to be implemented, video instructions with age-specific content plus hands-on training may improve learning for older adults.

## Background

For more than twenty years deep brain stimulation (DBS) is being used in patients with movement disorders, predominantly in refractory Parkinson's disease (PD) followed by dystonia and essential tremor (ET). More than 75.000 patients worldwide have undergone DBS ever since [1]. In the last couple of years, new manufacturers have entered the market and DBS is currently under investigation for indications such as major depression, obsessive-compulsive disorder or epilepsy [2]. All DBS systems include hand-held therapy controllers that patients obtain for home-use. With this technical device, patients are able to switch stimulation off and on, to in- or decrease it within a small range and to check the battery status of their implantable pulse generator (IPG). The latter is necessary in order to have invasive IPG exchange in time without therapy loss. Currently, the Medtronic Access controller is the most commonly used DBS patient controller. In practice, we observed that especially elderly patients refuse to use the controller or encounter some difficulties when they try to operate the device. However, to the best of our knowledge, no research has been done on the usability of DBS patient controllers.

In our recent work [2] we discussed DBS patient controllers in the context of ambient assisted living (AAL). We also argued that DBS therapy controllers, which are currently available on the European market, have major drawbacks in design and usability with regard to elderly patients. At an advanced age, the ability to correctly perceive high-frequency sounds or short-wave length colors (e.g. blue, violet) decreases. In addition, movement control and cognitive functions such as flexibility, attention or working memory decline in normal aging. These decrements are even more cumulated in older adults with chronic diseases such as Parkinson's disease [3-5]. Therefore, the design of DBS patient controllers should be very intuitive and congruent with patients' expectancies and mental models. Moreover, older adults have special needs when they are learning new technologies but perform as well as younger persons, if they receive appropriate training [6]. In the past, several groups demonstrated that instructional videos are effective training methods for older adults when it comes to learning new technologies. Mykityshyn et al. [7] reported that video instructions are more effective than text-based user manuals in subjects learning how to use a blood glucose meter. The video was filmed from the user's perspective. Elderly participants performed as accurate as their younger counterparts. Video-based instruction material was also effective in older adults who learned how to use a ticket vending machine [8]. The authors emphasize the importance of observational learning by video modeling to optimize outcomes.

Based on our previous work [2] and on the observation that in practice many elderly patients fear to use the Access therapy controller or mishandle it, we decided to investigate the usability of this device in elderly DBS patients that have been using the controller for several years. We also investigated the immediate and longer-term effects of different training modalities (interactive training and age-specific video material vs. interactive training alone).

## Methods

Patients were recruited from the Medical University of Vienna, Department of Neurosurgery and from the private practice of Dr. Oehlwein in Gera, Germany.

DBS patients age 55 plus were included in the study. We chose this context-bound lower age limit of at least 55 years following the MOBILATE study [9] according to which using new technology is not part of the daily life of people aged over 55. Further inclusion criteria were absence of dementia (MMSE score > than 23), Kinetra IPG longer than six months (so that patients have had the opportunity to get familiar with the patient controller) and written informed consent.

During the screening process, eight patients were found to have cognitive deficits and another eight patients refused to participate in the study, as they had too long distances to travel. Four other patients had to change their IPGs and subsequently received newer IPG models. These patients could therefore not be enrolled. Finally, a total of 41 patients with Parkinson's disease or essential tremor met inclusion criteria and also gave written informed consent.

During the follow-up four patients (two of each group) were lost, as they could not meet the deadline of the second assessment.

To avoid biases, the groups were matched according to age and score of the KUT questionnaire. Twenty participants were assigned to the experimental group. After having seen an age-specific instructional video, this group had to solve nine tasks (as described below) with the hand-held patient controller on a dummy IPG. For reasons of simplification this group hereinafter is referred to as "video group (VG)".

The other group ("control group", CG) consisted of 21 patients who performed on the dummy IPG without having seen the video-based training.

Any errors that patients made were documented and subsequently corrected so that all of them received interactive training. After six months all assessments on the dummy IPG were repeated in order to compare the effects of interactive training alone vs. combined with video-based training.

We produced an instructional video in which an elderly female patient with DBS acted as a model to show how to correctly use the Medtronic Access patient controller. We used video modeling to provide observational learning but also close ups from the user's perspective. In addition, a German voiceover commentary was used to support dual encoding. The video was 12 minutes long and after a short introduction, nine assignments were presented: 1) opening the lid of the controller, removing the battery and putting it back, 2) holding the controller at the IPG in the right direction, 3) switching on the IPG and naming the correct symbol at the backside of the controller, 4) turning off the IPG and finding the corresponding symbol at the backside, 5) checking the battery of the IPG and naming the symbol, 6) checking the battery of the patient controller and naming the symbol, 7) explaining where to change stimulation settings and the meaning of single and triple beeps (note: a single beep sounds when stimulation is increased by 0.1 V, a triple beep means that the upper or lower borders of the parameters have been reached), 8) increasing stimulation on both body sides by 0.1 V and explaining the meaning of single and triple beeps 9) decreasing stimulation on both body sides by 0.1 V and explaining the meaning of single and triple beeps Each lesson of the video started with a description of the learning content.

Struve & Wandke [8] propose that guided error training is more effective than error-free training in teaching older adults to use new technologies. However, we chose an error-free video version, as our patients were trained on a device that they have been using for several years. Thus, guided error training might have caused even more confusion in patients that are no novice users and that have already had the opportunity to make their own mistakes at home. The instructional methods for older adults followed the above-mentioned group (Table 1).

**Table 1:** Instructional Video

Characteristics	Instructional Design
Acoustics	Adjustment of volume according to individual needs

Vision	Use of high contrast and big font size

Cognitive load	Dual encoding of learning content, short sequences of modeling, consistent wording, short sentences, constant repetitions of learning content, attention guiding by using close ups, avoiding irrelevant information, learning content starts from easy to more difficult

Acceptance and self-efficacy	Emphasizing the benefits of properly using the controller, watching a patient who uses the controller with self-confidence (social cognitive learning)

Age specific characteristics of the instructional video (modified according to [8])

After completing the MMSE screening and questionnaires (see Table 2) the video group was watching the instruction material. Subsequently, their knowledge transfer was examined by using a dummy IPG. Patients were asked all of the nine assignments that the video group had seen in the training material. The control group was examined without having seen the training before. To diminish possible fear of contact, we chose to use an external model IPG instead of asking patients to apply their knowledge to their own, implanted pulse generators.

**Table 2:** Questionnaires

Baseline Assessments	Video Group Mean (SD)	Control Group Mean (SD)	*p*-values
Perceived self-competence in using technology	2.62 (1.29)	2.65 (1.25)	0.937

Acceptance of technology	2.91 (0.94)	3.04 (1.19)	0.698

Operability of the therapy controller	3.88 (0.88)	3.64 (1.20)	0.487

Fear of using the therapy controller	3.58 (1.23)	3.31 (1.43)	0.529

Active use of the therapy controller	4.05 (1.26)	4.19 (1.14)	0.739

FKK-SK (generalized self-concept of own abilities)	31.60 (SD 4.46)	30.42 (SD 6.71)	0.525

FKK-I (internal control orientation)	33.60 (SD 4.85)	34.52 (4.45)	0.528

KUT (control orientation with respect to technology use)	22.1 (SD 9.14)	23.45 (SD 8.38)	0.868

FLZ (quality of life)	370.22 (SD 43.12)	364.57 (SD 60.91)	0.733

At baseline both groups were comparable

We recorded the number of attempts and mistakes and measured the time to task completion. If patients did not know at all how to solve a technical problem, they were encouraged to try a different solution so that finally all patients performed the tasks correctly. To increase procedural knowledge, assignments were presented from easy to more difficult and in evolutionary steps, e.g. in assignment 7 patients were asked about the meaning of single and triple beeps. In assignment 8 and 9 the same questions occurred again, so that even patients that previously had failed to answer it correctly, were able to learn during performing.

When the IPG battery is about to deplete, the patient controller indicates that an invasive change is required, by displaying a flashing light during battery check. Depending on the individual stimulation parameters, patients have approximately one month time to receive a new IPG. In practice, we have frequently observed that patients missed this time frame, so we asked them about the frequency of use before and after the first assessment. Thus, patients were also asked about the active and passive frequency of use of the patient controller.

## Results

### Patients and questionnaires

Video and control group were comparable with respect to mean age, sex, year of IPG implantation, cognitive state, education, experience with technology in their working life and KUT score (control orientation with respect to technology use [10]) (see Table 3 and 4). All data were analyzed with PASW Statistics 18.0 for Mac.

**Table 3:** Patient characteristics

Baseline Characteristics & Assessments	Video Group n = 20 (PD = 16, ET = 4)	Control Group n = 21 (PD = 18, ET = 3)	*p*-value
Age in years	66.4 (SD 5.11)	65.90 (SD 5.47)	0.880

Sex	6 female, 14 male	5 female, 16 male	0.462

Year of first Kinetra implantation	2003	2004	0.726

MMSE [11]	28.3 (SD 0.73)	28.1 (SD 0.94)	0.347

Education	≤8 years (30%) 8-11 years (35%) ≥12 years (35%)	≤8 years (19%) 8-11 years (38.1%) ≥12 years (42.9%)	0.461

Technical Profession	Yes = 40%, No = 60%	Yes = 42.9%, No = 57.1%	0.552

Demographic characteristics of patients

**Table 4:** Frequency of use

Frequency of use: all patients	Battery check (patients)		Battery check (caregivers)		Changing stimulation settings (patient)		Changing Stimulation settings (caregiver)	
	
	pre	post	pre	post	pre	post	pre	post
Every day	6 (14.6%)	-	-	-	3 (7.3%)	2 (5.4%)	-	-

More often than once a week	2 (4.9%)	3 (8.1%)	-	-	1 (2.4%)	2 (5.4%)	-	-

Once a week	3 (7.3%)	7 (18.9%)	1 (2.4%)	1 (2.7%)	3 (7.3%)	-	-	-

More often than once a month	8 (19.5%)	5 (13.5%)	1 (2.4%)	1 (2.7%)	2 (4.9%)	1 (2.7%)	1 (2.4%)	1 (2.7%)

Once a month	3 (7.3%)	9 (24.3%)	-	-	3 (7.3%)	6 (16.2%)	-	-

Every 2-3 months	1 (2.4%)	6 (16.2)	-	1 (2.7%)	2 (4.9%)	4 (10.8%)	-	-

Every 4-6 months	5 (12.2%)	1 (2.7%)	2 (4.9%)	1 (2.7%)	5 (12.2%)	6 (16.2%)	-	2 (5.4%)

Less than every 4-6 months	13 (31.7%)	6 (16.2%)	37 (90.25)	33 (89.2%)	22 (53.7%)	16 (43.2%)	40 (97.6%)	34 (91.9%)

How often do patients or their caregivers check the IPG battery or change stimulation settings?

Based on the literature search a questionnaire was designed. After all patients had completed the questionnaire, reliability and explorative factor analysis revealed the following five scales: 1) perceived self- competence in using technology (Cronbach's α = 0.90), 2) acceptance of technology (Cronbach's α = 0.86), 3) Operability of the therapy controller (Cronbach's α = 0.78), 4) fear of using the therapy controller (Cronbach's α = 0.79) and 5) active use of the patient controller (Cronbach's α = 0.88). The questionnaire consisted of 22 items with a six point likert scale ranging from zero to five points, the total reliability was Cronbach's α = 0.91.

No group differences were found in locus of control (FKK-SK and FKK-I [12]) or in overall quality of life (FLZ [13]) (see Table 2), indicating that both groups were comparable. Six months after baseline assessment, no significant changes were found in the questionnaires.

### Use of the therapy controller

**Figure 1:**
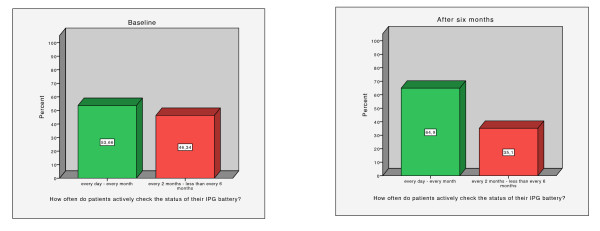
**Battery check**. Left: IPG battery check at baseline. Right: IPG battery check six months later (all patients).

The following graphs indicate that at baseline nearly half of the patients examined their battery status too rarely (every two months or less). By contrast, six months after the first assessment, about two thirds of the subjects checked the IPG battery in time (see Figure 1). However, probably due to the small sample size, this effect was not significant (Mc Nemar test, *p *= 0.388). Moreover, no significant results were found between video and control group (Mc Nemar test: VG: *p *= 0.687, CG: *p *= 0.687).

No significant between group effects were detected, when patients were asked about the active and passive use of the controller (see Table 4).

The results also revealed that caregivers are hardly involved in using the therapy controller. Both, at baseline and at the second assessment, the vast majority of patients stated that they are left on their own resources when it comes to checking the IPG battery. At both assessments, more than 90% of patients stated, that they have no caregiver support with respect to the use of the IPG.

### Assignments

**Figure 2:**
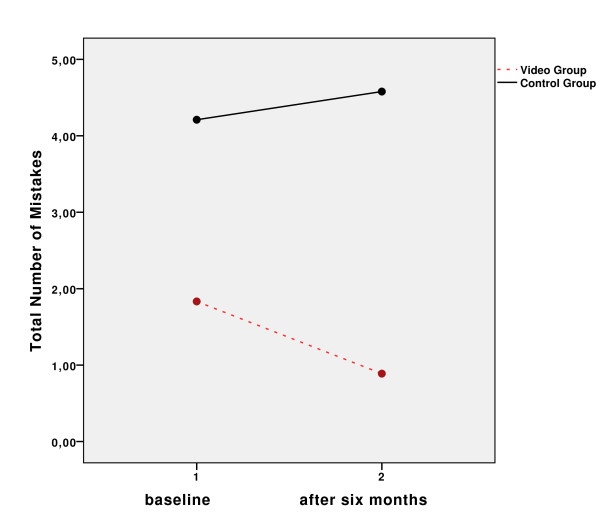
**Errors**. Differences in total number of mistakes in the course of time.

In the practical part of the study, the video group was significantly less error prone than the control group. After completing the video-based training, the mean error rate of the video group was 1.65 (SD = 1.81). The control group, however, made more mistakes (mean = 4.14, SD = 2.67). Six months after the first assessment this difference was still present. In the video group the mean error rate was 0.89 (SD = 1.02), while it was 4.58 in the control group (SD = 2.55). The mixed model ANOVA revealed significant interaction between the groups and time factor (F_(1;4,25)_, *p *= 0.047). Figure 2 shows the total number of mistakes by group at baseline and after six months respectively.

**Figure 3:**
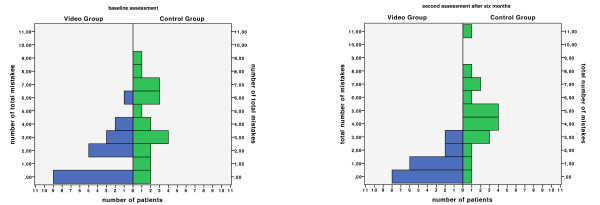
**Distribution of mistakes**. Distributions of mistakes between groups at baseline (left) and after six months (right).

Figure 3 displays the distributions of mistakes between groups at both points in time.

A closer look at the individual assignments reveals specific problems of the control group compared to the video group. The following table (Table 5) depicts significant and almost significant results of Mann and Whitney Tests:

**Table 5:** Specific problems

Tasks - Differences between groups at baseline (T1) and six months later (T2)	T1 Video Group Number of Attempts (SD)	T1 Control Group Number of Attempts (SD)	T1 *p*-value	T2 Video Group Number of Attempts (SD)	T2 Control Group Number of Attempts (SD)	T2 *p*-value
Ass.1: Opening the lid of the controller	1.30 (0.57)	1.81 (0.93)	0.51	1.17 (0.38)	1.95 (0.91)	0.01

Ass. 5: Identifying the icon of the IPG battery	1.20 (0.41)	1.57 (0.51)	0.02	1.11 (0.32)	1.42 (0.51)	0.11

Ass. 7: Knowing the meaning of a single beep	1.00 (0.00)	1.33 (0.48)	0.05	1.00 (0.00)	1.11 (0.32)	0.60

Ass. 7: Knowing the meaning of a triple beep	1.10 (0.31)	1.52 (0.51)	0.04	1.17 (0.38)	1.31 (0.48)	0.44

Mann-Whitney-Tests, specific problems at individual task

**Figure 4:**
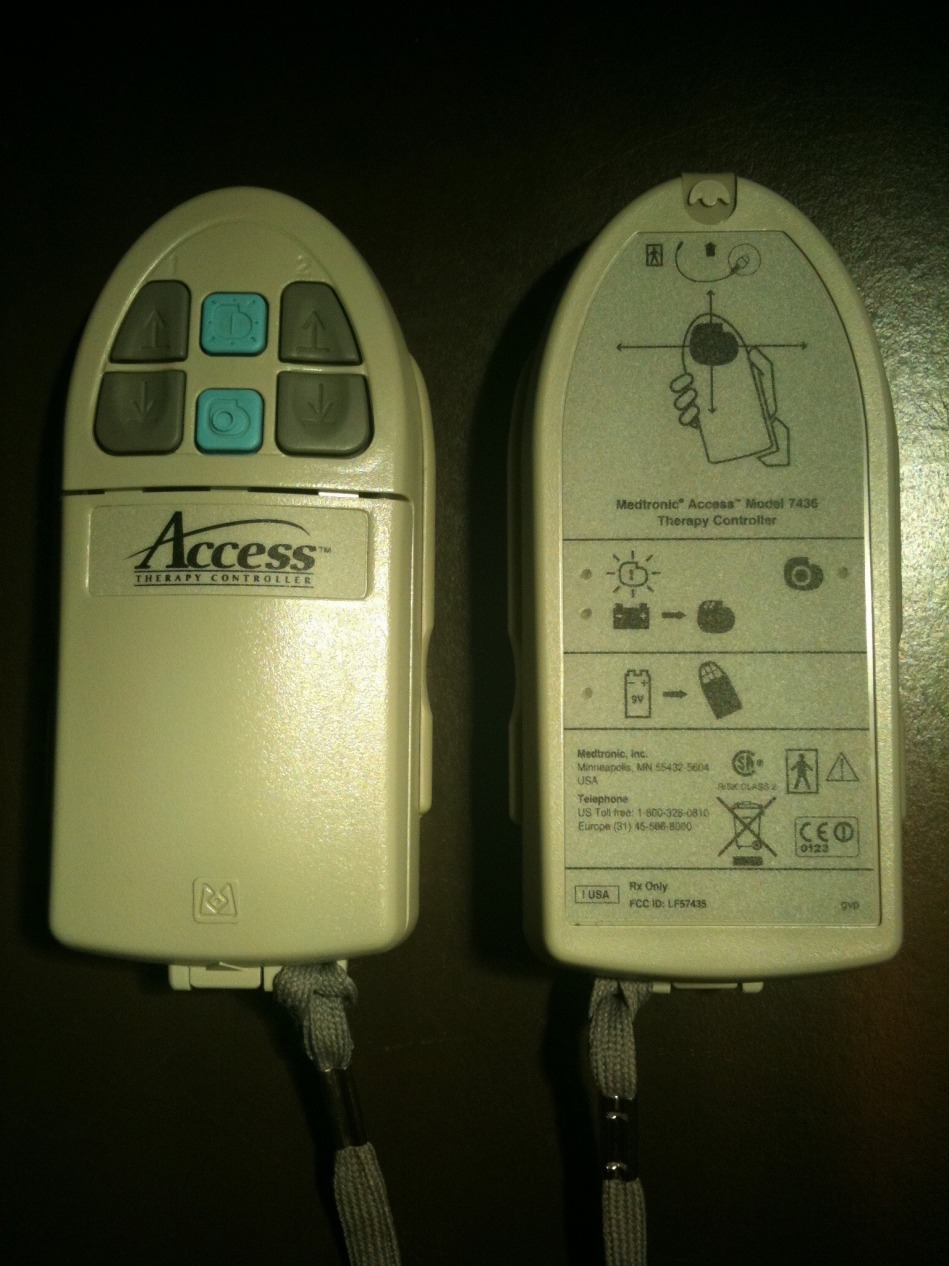
**Therapy controller**. Front- and backside of the therapy controller, the arrows mark the symbol for IPG and the circle contains the icon for IPG battery.

Right after watching the video-based training, patients of the video group outperformed patients of the control group in naming the type of the battery that is required for the patient controller and in recognizing the icon of the IPG battery on the backside of the controller. It shows a battery and an arrow pointing at the IPG symbol (see Figure 4).

Moreover, the video group was better in identifying the meanings of single and triple beeps in assignment 7. This difference, however, was absent in the following assignment 8 and 9 that also contained this question. An almost significant result was found in opening the lid of the patient controller. Six months after the baseline assessment, the performance of both groups was re-evaluated and significant differences were found in removing the lid and still in naming the type of battery of the therapy controller.

### Task completion time

In addition, the time to complete each task was measured from each subject. At the first assessment, differences between video and control group were only found in assignment 5: checking the IPG battery and identifying the IPG icon correctly (VG: mean = 22.45 sec, SD = 15.05; CG: mean = 33.29 sec, SD = 18.50; *p *= 0.07).

**Figure 5:**
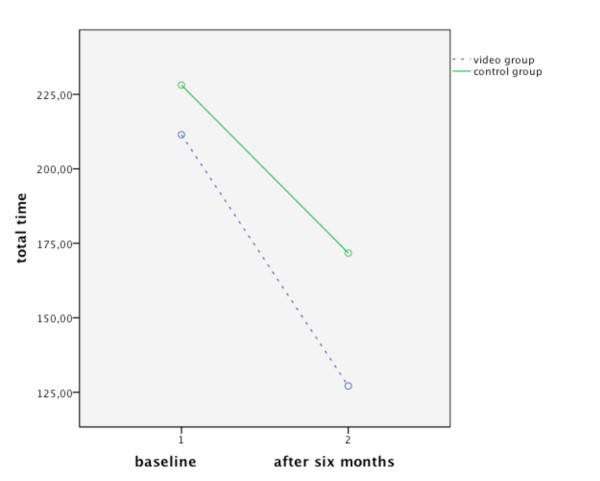
**Task completion time**. Changes in total time per group at baseline and after six months.

The overall time score did not differ significantly between groups at the first assessment (VG: mean = 203.3 sec, SD = 106.5; CG: mean = 219.14 sec, SD = 96.55; *p *= 0.62). Using a mixed model ANOVA, both groups were found to perform faster at the second assessment: F_(1,35) _= 26.133; *p *< 0.001. After six months the video group performed faster than the control group, albeit not significantly: F_(1;35) _= 1.032; *p *= 0.317 (see Figure 5).

## Discussion

The aim of the present study was to investigate usability aspects of DBS patient controllers and the impact of hands-on training with and without the combination of age-specific video instructions in elderly DBS patients with movement disorders. The most important results are as follows:

1) After having seen the video-based training, patients solved many tasks on the model IPG significantly better than the control group. Significant differences were found in opening the lid of the controller, naming the correct type of 9 V battery, identifying the icon of the IPG battery and knowing the meaning of single and triple acoustic signals.

2) A six months, the video group still outperformed the control group indicating that age-specific video instructions and hands-on training have more beneficial effects on usability aspects than interactive training alone.

3) The video group also performed faster than the control group at both assessments.

4) At the second assessment, the control group was faster than it was at baseline and but still made similar errors as before. The most common mistakes were opening the cover of the controller and knowing the meaning of the IPG battery. This implies that there were slight learning effects within the control group, probably induced by the presentation order of the interactive assessment with the dummy IPG at baseline. However, this difference was not significant compared to the effectiveness of the video-based training.

5) Patients have hardly any caregiver support when it comes to using the DBS controller.

6) In general, half of our patients used the therapy controller too infrequent for checking the battery status of the IPG at baseline. Six months later two thirds used it regularly. This difference was not significant, probably due to the small sample size.

The results show that patients, who have been using the device for several years, still have many difficulties and uncertainties. These results imply that hand-held patient controllers are not very intuitive and require improvement in usability aspects for end-users. However, we also demonstrated that the use of an age-specific video-based training has beneficial effects on the performance of patients. Right after watching the video, patients made fewer errors on a dummy IPG than patients who had not seen the video instructions. After a six months retention interval this difference was still present. Thus, appropriate training may lead to a more confident interaction with technical products, even if they do not entirely meet the needs of the end-users and even if this intervention did not have any influence on the patients' attitude towards the device.

Mykityshyn et al. [7] also found video instructions to be superior to text-based user manuals for this age group. Up until now, patients using the Access patient controller only receive a written user manual. Physicians personally explained the device to our patients at the end of their hospitalization after electrode placement or sometimes also if patients had specific questions during a follow-up visit. Apparently, this unsystematic approach was not enough. In the future, additional effective and age-specific video material and greater personal helpdesk may be preferable to support elderly patients to learn how to correctly use DBS controllers. Some clinics offer the support of specialized Parkinson's or DBS nurses to train the patients on the therapy controller. However, the availability of such specialized nurses is by far not everywhere ensured. Even if patients receive sufficient personal helpdesk by a DBS nurse at the clinic, video instructions may support them at home if questions arise. Operating the controller incorrectly may provoke acute physical interventions on the body and may lead to fear of use in those who do not feel entirely competent in using the device. Additional video material that can be watched over and over again may support and encourage patients to use the therapy controller in daily life, when there is no support from the clinic. This is especially valid for patients who live in remote areas and, as mentioned above, for those who have no access to DBS nurses.

Video modeling or observational learning may be crucial to improve the understanding of how to use a technical devices [8] but also to increase self-efficacy and self care and reduce anxiety in educating patients regarding treatment options [14]. As rechargeable DBS systems are being established on the market, usability aspects and appropriate training options become even more important because patients need to take a much more active part in their aftercare that patients with primary cell systems.

In our previous work [2] we have already pointed out the gerotechnological strengths and weaknesses of DBS patient controllers. With advanced age, decrements occur i.a. in the perception of short wave length colors such as blue. In the Medtronic Access controller, the on- and off-keys are very narrowly located to each other and have the same blue color, which makes confusion bound to occur. As on- and off-keys usually are presented in signal colors such as green or red, respectively, the blue coloring of the buttons goes against general mental models and expectancies [15]. Visual impairments that come with normal aging also require good contrast ratio on technical devices [15]. However, the contrast between the buttons and the grey housing of the controller is relatively poor (see Figure 4). This is also valid for contrast ratio of the icons of the buttons. Most importantly, the control group did not recognize the IPG icon. This symbol is depicted on the on- and off-keys as well as three times on the backside of the controller next to the control lights. The IPG icon may not be self-explanatory, as the majority of patients have never seen a pulse generator, which is implanted under their skin. Previous studies have shown that signs and symbols increase the speed of information processing, given that subjects have had the opportunity to learn their meaning [15].

As the controller has no display, patients have to count and remember the steps when they alter their stimulation settings, which may be a problem with respect to cognitive load. This is even more important considering the fact that many PD patients develop cognitive impairment in the course of their disease [16].

The Access therapy controller only communicates via high frequency sounds. The ability to perceive high frequencies also declines with normal aging [3]. Our results show, that the control group had difficulties to identify the meaning of a single and triple beep at baseline (assignment 7). Thus, after several years of using the controller, they did not entirely understand the only possible means of communication of the device. However, after six months there was no significant difference between groups anymore, probably due to task presentation that included various repetitions. After assignment 7, patients were asked to either increase or decrease the stimulation on the dummy IPG and again to identify the meaning of the emitted acoustic sounds. Hence, although patients of the control group had not seen the video-based training, they actively learned by the specific presentation order, albeit slower.

Compared to the video group, patients of the control group had greater difficulties in opening the cover of the therapy controller at both assessments. In practice, many patients did not find the correct release mechanism of the lid but tried to open it by squeezing on the both sides of the device. The release mechanism in its present form might go against expectancies and mental models of elderly patients.

The control group also had problems in identifying the correct name of the nine-volt battery that is used in the therapy controller. If the battery of the controller is low, patients need to change it themselves. On the backside of the therapy controller there is a control light with an icon showing a nine-volt battery. Thus, it is important for patients to know the meaning of it. The reason for the poorer performance of the control group may be due to the fact that nine-volt batteries are hardly ever used in hand-held devices such as TV remotes. Hence, common mental models and expectations may have been violated. The other group was confronted several times with this kind of battery in the video instructions and thus performed better.

Assuming that a depleting IPG battery is completely drained after more than a month, our results show that at baseline about half of the patients were at risk to experience sudden therapy loss and that caregivers barely use the controller. After six months the amount of all patients who checked the IPG battery in time increased up to two thirds. Probably due to small sample size, this difference was not significant. However, our data show that patients tend to use therapy controller on an irregular basis.

Our study has limitations due the relatively small series of patients. In the future more research should be done on larger patient samples and also for a longer period of time. Also the rater was not blinded. However, due to the given comparability of groups and the objective criteria at the nine assignments that the patients had to complete, the results seem to be robust.

Our results also show that patients change their neuromodulation settings very rarely. About half of the patients hardly ever change their parameters. If patients would feel more confident when operating the controller, they would probably make more often use of its advantages and adjust their settings according to their daily constitution. As it is not vital to adjust the stimulation settings on a regular basis, there should be other incentives like emotional design features to make using the controller more attractive to patients. Improved usability is even more important, considering that patients are left on their own when it comes to using the therapy controllers. The majority of the participants of our study stated that they have hardly any caregiver support.

Significant results were found in task completion time. Both groups significantly improved their performance at the second assessment compared to baseline. At both points in time, the video group was faster than the control group. This difference, however, was not significant, probably also due to small sample size. The learning effects of both groups indicate the positive impact of any kind of personal help desk, which should be provided in the clinics' routine.

## Conclusion

Older adults using DBS controllers for several years still have many difficulties in operating the device. The combination of an age-specific instructional video and active training on a dummy IPG yielded positive results, whereas active training alone was less effective. These results imply that elderly DBS patients benefit from age-specific training materials and personal help desk.

## Competing interests

The authors declare that they have no competing interests.

## Authors' contributions

IK: data collection, statistical analysis, interpretation and writing, IK-E: reviewing and writing, TC: reviewing and writing, FA: conception and design of manuscript and final approval of the version to be published. All authors read and approved the final manuscript.

## Pre-publication history

The pre-publication history for this paper can be accessed here:

http://www.biomedcentral.com/1471-2377/12/4/prepub
